# An exploratory study of nimotuzumab combined with toripalimab and chemotherapy for locally advanced tonsillar cancer

**DOI:** 10.3389/fimmu.2026.1890647

**Published:** 2026-07-14

**Authors:** Jingxuan Shi, Zhaoxiang Wang, Qianqian Zhao, Pengfei Liu, Leping Liang, Guanyin Chen, Dongjie He, Daqing Zhao

**Affiliations:** 1 Department of Otolaryngology–Head and Neck Surgery, Tangdu Hospital, The Fourth Military Medical University, Xi’an, Shaanxi, China; 2 School of Medicine, Northwest University, Xi’an, Shaanxi, China; 3 Department of Radiation Oncology, Tangdu Hospital, The Fourth Military Medical University, Xi’an, Shaanxi, China

**Keywords:** functional preservation, neoadjuvant therapy, nimotuzumab, surgical margin, tonsillar squamous cell carcinoma, toripalimab

## Abstract

**Background:**

Neoadjuvant immunotherapy/targeted therapy combined with chemotherapy shows encouraging activity for oropharyngeal squamous cell carcinoma (OPSCC). However, evidence specific to tonsillar squamous cell carcinoma (TSCC) remains limited, particularly regarding the systematic relationships among radiological response, pathologic response, margin control, and voice and swallowing functional outcomes.

**Methods:**

This single-arm, single-center study included patients with pathologically confirmed TSCC diagnosed between January 2024 and June 2025. Patients received triplet neoadjuvant therapy consisting of nimotuzumab, toripalimab, nab-paclitaxel, and carboplatin every 3 weeks for 2 cycles, followed by transoral radical tonsillectomy combined with radical cervical lymph node dissection. Radiologic response was assessed using Response Evaluation Criteria in Solid Tumors version 1.1 (RECIST 1.1) and volumetric magnetic resonance imaging (MRI). Pathologic response, margin status, perioperative safety, adjuvant treatment, and functional outcomes were evaluated.

**Results:**

Twenty patients achieved clinicopathologic downstaging after surgery. The primary-site pCR rate was 65.0%, nodal pCR was 60.0%, and overall ypT0N0 pCR was 45.0%. The objective radiological response (ORR) rate of the primary lesion was 100%, and 9 patients (45%) achieved a complete radiological response. Mean primary-tumor and dominant-node volume reductions were 76.18% and 72.59%, respectively. Both pre- and postneoadjuvant margins were negative in all patients, yielding R0 resection in all cases. Functional scores improved after neoadjuvant therapy, worsened transiently during radiotherapy, and generally recovered during follow-up.

**Conclusions:**

Nimotuzumab plus toripalimab and chemotherapy produced high radiological and pathological response rates with reliable margin control in patients with locally advanced TSCC. Postneoadjuvant boundary assessment with intraoperative pathologic verification may support individualized resection planning, but routine resection of the primary lesion and cervical lymph node dissection remain necessary. Longer follow-up and prospective validation are required to confirm these results.

**Clinical trial registration:**

## Introduction

OPSCC accounts for approximately one-quarter to one-third of all head and neck squamous cell carcinomas (HNSCCs), and of these, tonsillar squamous cell carcinoma (TSCC) is the most common subtype (1). The palatine tonsil is mucosa-associated lymphoid tissue located in the tonsillar fossa, and together with the adenoids, lingual tonsils, and lateral pharyngeal bands, forms Waldeyer’s ring, which functions in local immune defense in the head and neck (2, 3). The palatine tonsil lies adjacent to the soft palate, anterior to the base of the tongue, and inferior to the laryngeal region. Although traditional radical treatment for oropharyngeal squamous cell carcinoma (OPSCC) can achieve a certain degree of local control, it is often accompanied by problems such as dysphagia, phonatory abnormalities, nasogastric tube dependence, or tracheostomy. The way in which a balance between tumor control and functional preservation can be achieved remains a therapeutic challenge (4, 5).

TSCC is often diagnosed at a locally advanced stage, and current guideline-recommended treatment still consists of definitive chemoradiotherapy or surgery plus adjuvant chemoradiotherapy. Notably, the American Joint Committee on Cancer (AJCC) 8th edition downstages p16-positive OPSCC; therefore, although some cases are classified as stage I/II, when the burden of the primary lesion and cervical disease as well as the anticipated need for multimodality treatment are considered, these cases still represent a resectable TSCC population with relatively high treatment complexity. Human papillomavirus (HPV)-negative OPSCC is traditionally closely associated with tobacco and alcohol exposure, and compared with that of HPV-positive OPSCC, its prognosis is significantly worse. A clinical trial revealed that the 3-year overall survival (OS) and progression-free survival (PFS) rates in HPV-negative patients were only 57.1% and 43.4%, respectively, whereas the 3-year OS in high-risk patients stratified by HPV status and smoking exposure was only 46.2% (5–7). Although the National Comprehensive Cancer Network (NCCN) guidelines recommend chemotherapy combined with immunotherapy/targeted therapy for recurrent/metastatic and unresectable HNSCC, reports that address the efficacy, complications, and prognosis of neoadjuvant therapy in patients with tonsillar cancer as a single disease entity remain limited (8).

Although traditional radical treatment for oropharyngeal squamous cell carcinoma (OPSCC) can achieve a certain degree of local control, it is often accompanied by challenges such as dysphagia, phonatory abnormalities, nasogastric tube dependence, or tracheostomy. The way in which a balance between tumor control and functional preservation can be achieved remains a therapeutic challenge (4, 5).

In recent years, the use of neoadjuvant chemotherapy combined with immunotherapy and targeted therapy has become a research hotspot, with the goal of downstaging and shrinking tumors, improving resectability, further prolonging survival, and establishing conditions for subsequent treatment deintensification. Neoadjuvant strategies for OPSCC have achieved high pathologic complete response (pCR)/major pathologic response (MPR) rates and have shown potential advantages in the preservation of swallowing and speech function (5, 8). However, for TSCC as a single subtype, systematic reporting on the complete evidence chain surrounding ‘radiologic response–pathologic response–margin safety–surgical benefit–functional preservation’ is still lacking.

On this basis, the present exploratory study focused on TSCC as a single disease entity and used a triplet neoadjuvant regimen consisting of chemotherapy + an anti-epidermal growth factor receptor (EGFR) monoclonal antibody + an immune checkpoint inhibitor (ICI) followed by sequential surgery, with the pCR rate as the primary endpoint. Particular emphasis was placed on pre- and postneoadjuvant margin status, radiological response, quality of life, voice and swallowing function, and perioperative safety; in addition, the feasibility of treatment deintensification was further evaluated on the basis of postoperative pathologic risk stratification. The aim of this study was to address whether the triplet regimen could induce a high proportion of deep pathologic responses within a limited number of cycles and whether R0 resection could be achieved with a smaller extent of resection to improve patients’ quality of life.

## Materials and methods

### Study design and participants

This was a single-center, exploratory study. Patients with TSCC who presented to the Department of Otolaryngology of the Second Affiliated Hospital of Air Force Medical University between January 2024 and June 2025 and who underwent standardized treatment evaluation were included.

### Inclusion and exclusion criteria

The inclusion criteria were as follows: TSCC that was pathologically confirmed by biopsy; clinically assessed resectable disease; no evidence of distant metastasis on imaging. The exclusion criteria were nonsquamous cell carcinoma; unresectable tumor; distant metastasis; previous treatment for head and neck tumors; neck surgery, radiotherapy, or systemic therapy; and prior treatment with immune checkpoint inhibitors or anti-EGFR targeted agents. Before treatment, all patients underwent systematic physical examination, medical history collection, and multidisciplinary evaluation.

### Ethics approval

This study was registered at ClinicalTrials.gov (NCT07353723) and was approved by The Ethics Review Committee of Tangdu Hospital, The Fourth Military Medical University. All patients provided written informed consent. The use of clinical data and tumor specimens during the study complied with Institutional Ethical Standards and Institutional Review Board (IRB) requirements.

### Treatment protocol

All patients received nimotuzumab (400 mg), toripalimab (240 mg), nab-paclitaxel (260 mg/m²), and carboplatin (250 mg/m²), every 3 weeks. Two cycles were planned before surgery. During treatment, biochemical parameters, imaging data, and treatment-related adverse events (TRAEs) were recorded. After the planned neoadjuvant therapy was completed, imaging reevaluations (neck computed tomography (CT), contrast-enhanced neck magnetic resonance imaging (MRI), positron emission tomography–computed tomography (PET-CT), and cervical lymph node ultrasound) and electronic nasopharyngolaryngoscopy were performed. Surgical plans were formulated according to changes in tumor boundaries observed on imaging and endoscopy.

Surgery was performed 4 weeks after the completion of neoadjuvant therapy and consisted of transoral radical resection of the tonsillar cancer combined with radical cervical lymph node dissection. Bilateral cervical lymph node dissection was performed when the tumor crossed the midline. A histological margin of ≥ 5 mm was regarded as a conventional clear margin when assessable (9). To evaluate margin safety, intraoperative multipoint frozen-section sampling was performed using tissue from the tonsillar surgical bed with reference to two predefined margin concepts: the preneoadjuvant margin, corresponding to the pretreatment tumor extent reconstructed from baseline endoscopy, and the postneoadjuvant margin, corresponding to the residual tumor extent defined by postneoadjuvant endoscopy. Multipoint margin evaluation was completed intraoperatively to further verify margin safety and the controllability of the extent of resection.

### Clinical outcomes, pathology definitions, and follow-up

#### Radiologic and pathologic evaluation

Oropharyngeal cancer staging was based on the AJCC 8th edition TNM staging system, and radiological response was classified according to the Response Evaluation Criteria in Solid Tumors version 1.1 (RECIST 1.1) as partial response (PR) or complete response (CR) (10). Volumetric assessment was based on neck MRI measurements of the primary tumor and metastatic cervical lymph node lesion volumes, using ITK-SNAP (v3.8.0) for manual delineation and automated volume calculation. Imaging examinations were performed before the initiation of neoadjuvant chemotherapy and before surgery.

Resected primary lesions and cervical lymph nodes were subjected to routine histopathological examination. pCR was defined as no residual tumor at the primary site and no evidence of residual nodal disease in the cervical lymph nodes (ypT0N0). Clinicopathologic downstaging was defined as a lower overall pathological stage than the overall clinical stage at the start of NAC according to the AJCC 8th edition.

#### Postoperative treatment pathway

Investigators determined whether to administer adjuvant therapy or perform active surveillance according to the patient’s initial clinical stage, radiological and pathological response to neoadjuvant therapy, and the presence of adverse pathological features.

Criteria for postoperative adjuvant therapy included positive surgical margins or pathological high-risk factors (perineural invasion, lymphovascular invasion, extranodal extension, or multiple metastatic lymph nodes) and treatment with intensity-modulated radiotherapy (IMRT) at a total dose of 50–60 Gy according to the risk factors present. If pCR was achieved postoperatively, active surveillance or maintenance immunotherapy (240 mg of toripalimab intravenously injected once every 3 weeks for 1 year) could be adopted according to overall risk.

#### Follow-up and functional assessment

Functional outcomes were assessed throughout treatment and follow-up. Quality of life was assessed using the University of Washington Quality of Life Questionnaire version 4.0 (UW-QOL V4.0), swallowing function was assessed using the MD Anderson Dysphagia Inventory (MDADI) and the Eating Assessment Tool-10 (EAT-10), and voice function was assessed using the Voice Handicap Index (VHI) (11–15). Assessments were performed at baseline, after neoadjuvant therapy, during adjuvant radiotherapy when applicable, and during postoperative follow-up. Perioperative complications, including postoperative hemorrhage, nasogastric tube dependence, tracheostomy, neck infection, chyle leak, neurologic dysfunction, and voice/swallowing dysfunction, were also recorded.

### Study endpoints and statistical analysis

The primary endpoint was overall pathologic complete response (pCR, defined as ypT0N0). Secondary endpoints included pathologic response of the primary site and cervical lymph nodes, margin status, radiological response, clinicopathologic downstaging, postoperative adjuvant treatment pathways, perioperative safety, and changes in quality of life and swallowing/voice function. Categorical variables are described as frequencies (percentages). Continuous variables are presented as means with ranges or, where appropriate, as means ± standard deviations or medians with interquartile ranges. Between-group comparisons were performed using the chi-square test or Fisher’s exact test for categorical variables and the independent-samples t test or Mann-Whitney U test for continuous variables. Repeated UW-QOL V4.0, MDADI, EAT-10, and VHI scores were analyzed using linear mixed-effects models or mixed models for repeated measures; if model assumptions were not met, the Friedman test and Wilcoxon signed-rank test with multiple-comparison correction were used.

## Results

### Patient disposition and baseline characteristics

A total of 24 patients met the initial screening criteria. Of these, 2 patients refused surgery after neoadjuvant therapy (and ultimately received sequential radiotherapy after 6 cycles of chemotherapy) whereas 2 patients received only 2 cycles of neoadjuvant therapy for personal reasons (1 withdrew from the study because of a cerebrovascular accident, and 1 voluntarily withdrew). The remaining 20 patients completed sequential surgery after neoadjuvant therapy and were included in the final analysis.

The final analysis included 20 patients with TSCC who received triplet neoadjuvant therapy and underwent surgery (**Table 1**). The mean age was 56.9 years, and 13 patients (65.0%) were male. Nineteen patients (95.0%) had p16-positive tumors; all tumors showed positive EGFR expression, and most demonstrated at least weak positivity. Programmed death-ligand 1 (PD-L1) combined positive score (CPS) stratification revealed 5 patients (25.0%) with a CPS that was ≥ 1 but < 10, 6 patients (30.0%) with a CPS ≥ 10 but < 20, and 9 patients (45.0%) with a CPS ≥ 20. The proportions of patients with and without a history of smoking/alcohol use were comparable in this study.

**Table 1:** Patient characteristics.

Variables	Number of patients	%
Age (mean ± SD, years)	56.9 ± 7.62 (50–78)	–
Sex		
Male	13	65
Female	7	35
p16		
Positive	19	95
Negative	1	5
EGFR		
Weak positive (+/-)	1	5
+	14	70
++	3	15
+++	2	10
PD-L1 combined positive score		
1 ≤ CPS < 10	5	25
10 ≤ CPS < 20	6	30
CPS ≥ 20	9	45
Tobacco		
Yes	10	50
No	10	50
Alcohol		
Yes	9	45
No	11	55
Tumor side		
Left	9	45
Right	11	55

Data are presented as n (%) unless otherwise indicated. Age is presented as the mean ± SD (range). CPS, combined positive score.

### Treatment completion and safety

All 20 patients underwent surgery after the completion of neoadjuvant therapy. Among them, 16 patients (80.0%) completed the planned 2 cycles of neoadjuvant therapy, whereas 4 patients received 4 cycles because imaging and endoscopic evaluation after cycle 2 indicated insufficient tumor shrinkage to meet the conditions for R0 resection, after which all 4 patients were reevaluated and successfully underwent surgery (16). All the patients completed neoadjuvant therapy successfully, and no surgery was terminated because of adverse neoadjuvant-treatment-related reactions. The TRAEs were manageable overall (**Table 2**). The most common adverse events were alopecia (20 patients), decreased appetite/anorexia (10), fatigue (10), leukopenia (10, all grades 1–2), constipation (7), and anemia (6, all grades 1–2). One patient experienced grade ≥ 3 immune-related pneumonitis, improved after anti-infective and steroid therapy, discontinued targeted and immunotherapy agents, completed conventional chemotherapy, and underwent surgery.

**Table 2:** Treatment-related adverse events (TRAEs).

Adverse events	Patients (N = 20)		
	Grade 1	Grade 2	Grade 3
Alopecia	20		
Decreased appetite or anorexia	10		
Leukopenia	7	3	
Fatigue	10		
Constipation	7		
Anemia	4	2	
Rash	2	2	
Vomiting	3		
Immune-related pneumonitis			1

Blank cells indicate that no event of the corresponding grade was recorded.

### Stage changes and radiologic responses

**Figure 1:**
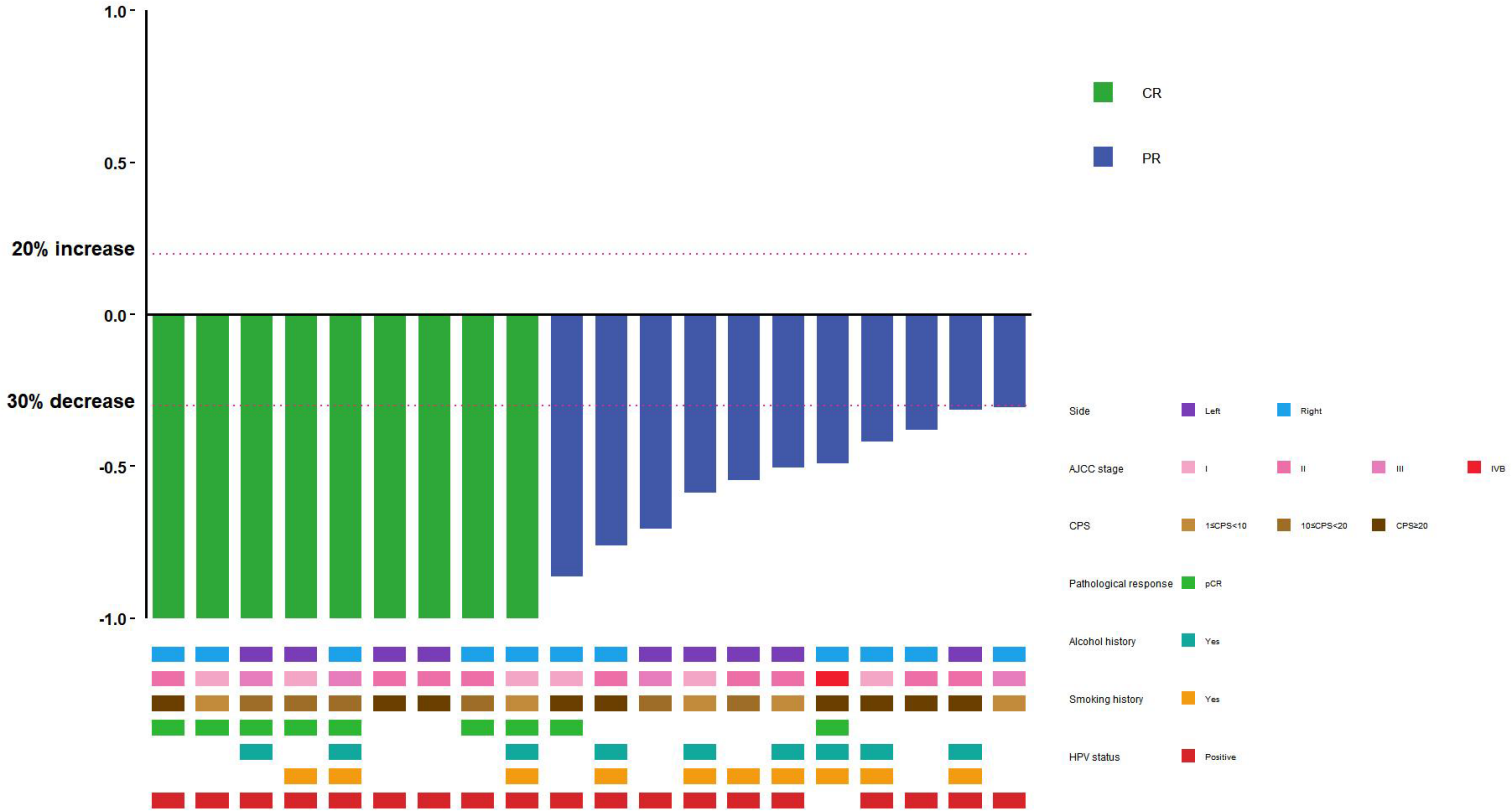
Tumor response in the 20 patients. Waterfall plot of the percent change in tumor response among the 20 patients. The green bars indicate the CR, and the blue bars indicate the PR; the annotation tracks display the side, AJCC stage, CPS group, pathologic response, alcohol history, smoking history, and HPV status.

The postoperative overall pathological stage was lower than the pretreatment overall clinical stage in all patients, yielding a clinicopathologic downstaging rate of 100.0% (**Tables 3**, **4**). Radiologically, all the primary lesions achieved an objective response (**Figure 1**), and 9 patients (45.0%) achieved a radiological complete response (CR), while the remaining 11 (55%) achieved partial response (PR). Volumetric analysis showed that the mean primary tumor volume decreased from 16.09 cm³ to 2.92 cm³, with a mean reduction of 76.18%, the mean dominant metastatic lymph node volume decreased from 16.00 cm³ to 1.92 cm³, with a mean reduction of 72.59% (**Table 5**). Overall, neoadjuvant therapy resulted in marked volume reduction in both primary tonsillar lesions and dominant cervical metastatic lymph nodes.

**Table 3:** Clinical classification and staging before neoadjuvant therapy.

Clinical classification	N0	N1	N2	N3	Stage	Count
T0					I	6
T1					II	9
T2		8			III	4
T3	1	6			IV	1
T4	1	3		1		

The table presents the distribution of clinical T and N categories together with overall stage and case count before neoadjuvant therapy. Blank cells indicate zero cases.

**Table 4:** Postsurgical pathologic classification and staging.

Pathologic classification	ypN0	ypN1	ypN2
ypT0	9	4	
ypT1	4	2	1
ypT2			
ypT3			
ypT4			

The table presents the distribution of pathologic T and N categories after treatment. Blank cells indicate zero cases.

**Table 5:** Image reduction ratio of tumor volume after neoadjuvant therapy.

Tumor volume	Preneoadjuvant therapy (cm³)	Postneoadjuvant therapy (cm³)	Reduction ratio of tumor volume (%)
Mean primary-tumor volume	16.09 (3.37–48.71)	2.92 (0.32–14.30)	76.18% (19.66%–96.87%)
Mean metastatic-node volume (dominant node)	16.00 (0–107.42)	1.92 (0–7.00)	72.59% (0%–98.38%)

Values are presented as the mean (range).

### Pathologic response, margin status, and surgical outcomes

Pathologic response results are shown in **Table 6**. The overall pCR rate was 45.0% (9/20, 95% CI: 23.1%–68.5%), the primary site pCR rate was 65.0% (13/20, 95% CI: 40.8%–84.6%), and the nodal pCR rate was 60.0% (12/20, 95% CI: 36.1%–80.9%). All patients underwent radical resection of tonsillar cancer combined with radical cervical lymph node dissection. Both the pre- and postneoadjuvant margins were negative in all patients. All patients resumed oral intake, and nasogastric tubes were removed after surgery. No hemorrhage, tracheostomy, neck infection, chyle leak, or obvious neurologic dysfunction was observed during hospitalization.

**Table 6:** Clinical to pathological response following treatment.

Outcome (n = 20)	n (%)
Overall pCR	9 (45%)
Tumor response at primary site	
Complete response	13 (65%)
Partial response	7 (35%)
Nodal response	
Complete response	12 (60%)
Partial response	8 (40%)
Intraoperative margin status	
Preneoadjuvant margins negative	20 (100%)
Postneoadjuvant margins negative	20 (100%)
Clinical-to-pathologic downstaging	
Yes	20 (100%)
No	0
Recurrence	0%
Major postoperative complications	0%
Death	0%

pCR denotes ypT0N0. Percentages are calculated with n = 20.

CPS-stratified analysis is presented in **Table 7**. Overall ypT0N0 pCR rates were 40.0% (2/5), 66.7% (4/6), and 33.3% (3/9) in the 1 ≤ CPS < 10, 10 ≤ CPS < 20, and CPS ≥ 20 groups, respectively. The corresponding radiological CR rates were 40.0%, 66.7%, and 33.3%; ORR was 100% in all three groups. CPS category was not significantly associated with overall pCR or radiological response depth.

**Table 7:** Pathological and radiological responses according to PD-L1 CPS category.

PD-L1 CPS category	Patients, n	Overall pCR, n (%)	Radiological CR, n (%)	Radiological PR, n (%)	ORR, n (%)
1 ≤ CPS < 10	5	2 (40.0%)	2 (40.0%)	3 (60.0%)	5 (100%)
10 ≤ CPS < 20	6	4 (66.7%)	4 (66.7%)	2 (33.3%)	6 (100%)
CPS ≥ 20	9	3 (33.3%)	3 (33.3%)	6 (66.7%)	9 (100%)
Overall	20	9 (45.0%)	9 (45.0%)	11 (55.0%)	20 (100%)

pCR denotes ypT0N0; CR, complete response; PR, partial response; ORR, objective response rate.

### Postoperative adjuvant treatment and functional outcomes

#### Postoperative adjuvant treatment

Postoperative adjuvant therapy was determined according to pathologic risk stratification. Among the 20 patients, 4 underwent active surveillance, 2 received maintenance immunotherapy, 11 received radiotherapy because residual disease was confirmed in the resected tonsil and/or cervical lymph nodes, and 3 received radiotherapy combined with immunotherapy because of the presence of high-risk pathological factors.

#### Functional outcomes

**Figure 2:**
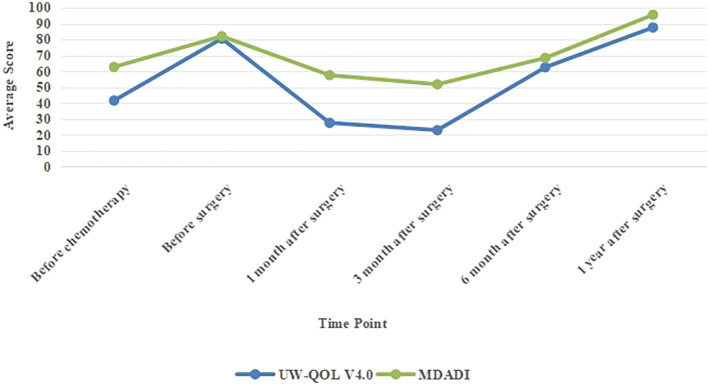
Changes in quality of life and swallowing-related scale scores over time. Serial line plot of UW-QOL V4.0 and MDADI scores across the prespecified assessment time points.

**Figure 3:**
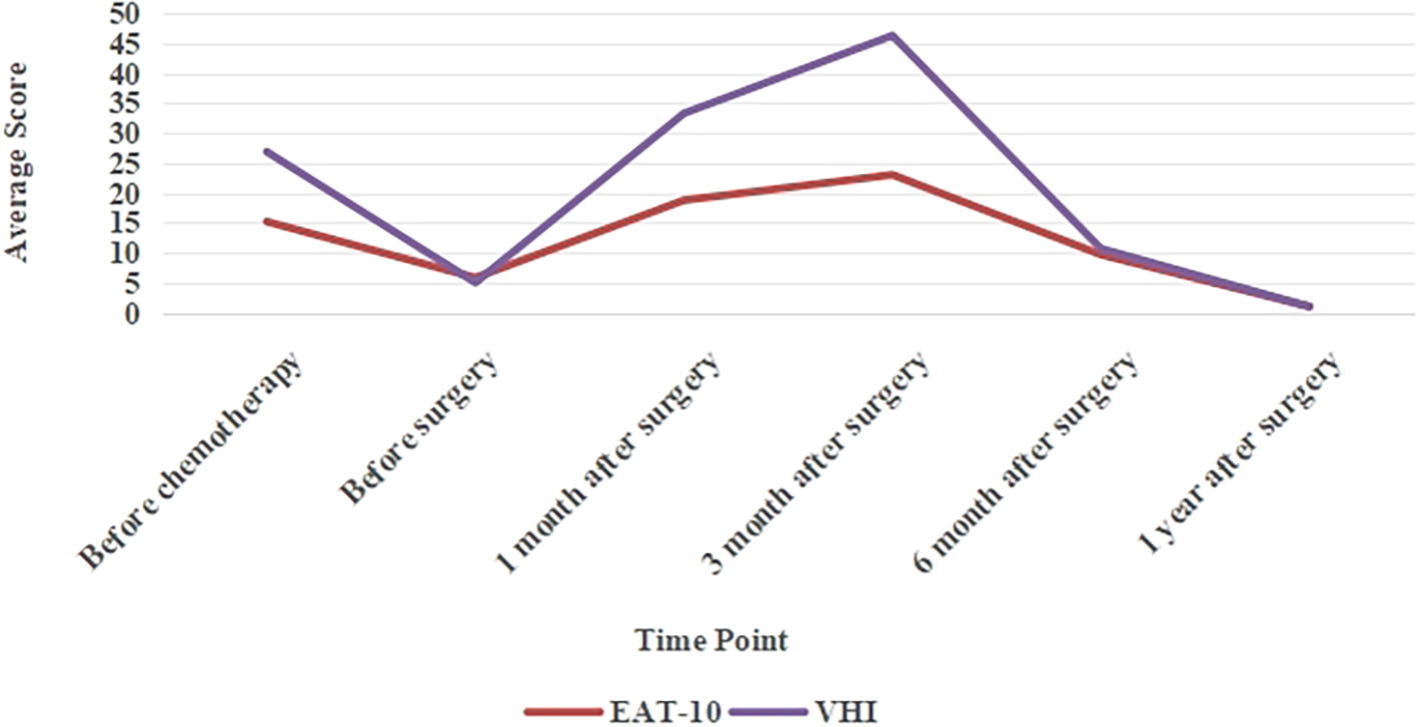
Swallowing and voice function scale over time. Serial line plot of EAT-10 and VHI scores across the prespecified assessment time points.

With respect to functional outcomes, the quality of life (UW-QOL V4.0) and MDADI scores both improved over the baseline after neoadjuvant therapy; in contrast, these scores decreased significantly after patients entered the radiotherapy phase, reached a nadir 3 months after radiotherapy, and then gradually recovered by 6 months after radiotherapy, approaching or returning to normal levels 1 year after surgery (**Figures 2**, **3**). With respect to swallowing function, EAT-10 indicated mild dysphagia before treatment, marked improvement after neoadjuvant therapy, short-term worsening after radiotherapy, and subsequent gradual recovery. With respect to voice function, the VHI showed mild baseline abnormalities, with the most marked improvement observed after neoadjuvant therapy, short-term deterioration seen after radiotherapy, and recovery to the normal range 6 months to 1 year after radiotherapy.

## Discussion

The combination of a PD-1-based immune checkpoint inhibitor with systemic therapy has become an important treatment strategy in recurrent/metastatic head and neck squamous cell carcinoma, leading to increased attention towards incorporating immunotherapy into neoadjuvant treatment for locally advanced disease. However, most neoadjuvant studies enroll mixed head and neck subsites, and evidence focusing specifically on tonsillar squamous cell carcinoma (TSCC) remains limited. This gap was addressed in the current study by evaluating neoadjuvant nimotuzumab, toripalimab, nab-paclitaxel, and carboplatin followed by surgery in patients with TSCC, with an emphasis on radiologic response, pathologic response, margin safety, perioperative feasibility, and functional outcomes.

Favorable short-term efficacy signals were observed. The overall ypT0N0 pCR rate was 45.0%, with primary-site pCR and cervical metastatic lymph node pCR rates of 65.0% and 60.0%, respectively. All primary lesions achieved an objective radiological response, including radiological CR in 45.0% of patients, and the mean primary-tumor and dominant-node volume reductions exceeded 70%. Importantly, both pre- and postneoadjuvant margins were negative in all patients, and all patients proceeded to surgery without neoadjuvant treatment toxicity preventing resection. These findings suggested that the regimen might not only deepen the early tumor response but also provide a basis for subsequent individualized surgical planning. Compared with previous neoadjuvant immunotherapy studies, these results help define the position of this three-class strategy. Most early trials enrolled heterogeneous populations of resectable head and neck squamous cell carcinoma (HNSCC) across multiple subsites. Neoadjuvant PD-1 monotherapy has been feasible in resectable HNSCC, but deep pathological responses are usually limited; CheckMate 358 showed pathologic responses after nivolumab, whereas pCR was uncommon and differed by HPV status. KEYNOTE-689 demonstrated that perioperative PD-1 inhibition improved event-free survival in resectable locally advanced HNSCC across multiple anatomic subsites, although the pCR rate within a short treatment window remained limited (17). Chemoimmunotherapy may enhance early tumor clearance, as shown by Wu et al. (18), who reported an ORR of 89.6% and a pCR rate of 55.6% with camrelizumab plus nab-paclitaxel and cisplatin in a mixed HNSCC cohort. OPSCC-focused studies such as OPTIMA II and E1308 also reported marked radiologic responses (19, 20). Unlike these studies, the present analysis focused on TSCC as a single disease entity and linked radiologic response with pathologic response, margin safety, surgical feasibility, and functional recovery.

R0 resection remains the basic oncologic goal, but the tonsillar region is anatomically narrow and closely associated with swallowing, phonation, and airway functions. Expanding the resection range may improve local control but can increase the risk of aspiration, nasogastric tube dependence, tracheostomy, and voice dysfunction; overly narrow margins may increase local recurrence and require more intensive postoperative treatment. Therefore, the surgical question after neoadjuvant therapy is not merely how much the tumor has shrunk, but whether the posttreatment boundary can safely guide resection (9, 21, 22).

Hinni et al. (23) and Tirelli et al. (24) used intraoperative narrow-band imaging in combination with TORS to assess margins in oropharyngeal cancer and adopted intraoperative piecemeal release/resection. Compared with traditional en bloc resection plus point sampling for pathology, intraoperative frozen-section margin assessment after piecemeal resection yielded higher specificity and sensitivity. Considering this background, the present study focused on the reevaluation of the surgical boundary after neoadjuvant therapy, which is more meaningful to real-world TSCC clinical decision-making than simply reporting the ORR. The present study verified the safety of the resection range pathologically by using predefined preneoadjuvant and postneoadjuvant margins together with intraoperative multipoint frozen-section assessment. Both the pre- and postneoadjuvant margins were negative in all patients, and R0 resection was achieved in all cases. This suggests that after a substantial treatment response has been achieved, the extent of resection may be determined according to the posttreatment tumor boundary in selected patients, provided that strict pathologic verification is ensured.

Radiologic response, however, should not be regarded as equivalent to histologic clearance. Mean primary and nodal volume reductions were substantial, but radiologic shrinkage does not necessarily indicate synchronous contraction of the microscopic tumor boundary or nodal sterilization. This characteristic is especially relevant in cervical disease, where the metastatic lymph node pCR rate was 60.0%, yet microscopic residual disease may persist despite favorable imaging findings, and residual imaging findings may also overestimate viable disease. Therefore, for patients with pathologically proven cervical metastases before treatment, neck dissection remains important for locoregional control at the present stage.

EGFR is frequently overexpressed in HNSCC and contributes to tumor growth, invasion, and metastasis (25). Anti-EGFR monoclonal antibodies combined with PD-1 inhibitors have demonstrated promising activity and acceptable safety in recurrent/metastatic HNSCC (26–29). Chemotherapy can reduce tumor burden and induce immunogenic cell death, thereby promoting tumor-antigen release and immune recognition (30–33). Anti-EGFR monoclonal antibodies activate NK cells through Fcγ receptor-mediated antibody-dependent cellular cytotoxicity, linking innate and adaptive immunity (25, 34, 35). PD-1 blockade may then sustain the activated antitumor T-cell response. Thus, the regimen is intended to act on a continuous process of tumor reduction, antigen release, immune amplification, and effector maintenance.

Previous evidence has demonstrated that PD-L1 CPS is associated with differential treatment outcomes in recurrent or metastatic head and neck squamous cell carcinoma. In the updated analysis of KEYNOTE-048, pembrolizumab-based treatment improved overall survival in the prespecified CPS ≥ 20 and CPS ≥ 1 populations, with a numerically greater benefit from pembrolizumab monotherapy observed in the CPS ≥ 20 population (29). In this study, an exploratory analysis was conducted to assess the potential association between PD-L1 CPS and treatment response. No statistically significant association was identified between CPS category and either overall pCR or radiological response depth. Given the small cohort, the exploratory nature of the analysis, and the potential influence of baseline tumor burden, p16 status, and treatment exposure, these findings do not establish PD-L1 CPS as a predictive biomarker for this neoadjuvant regimen. Larger prospective cohorts are required to clarify whether PD-L1 expression can identify patients most likely to benefit from this neoadjuvant strategy.

The safety profile of the current regimen was manageable. Most patients completed the planned 2 cycles of neoadjuvant therapy, and the four patients requiring extended treatment proceeded to surgery after reevaluation. No surgery was cancelled because of neoadjuvant-treatment toxicity. One patient developed grade ≥ 3 immune-related pneumonitis, improved after anti-infective and steroid therapy, discontinued targeted and immunotherapy agents, completed chemotherapy, and later underwent surgery. These findings support the perioperative feasibility of the regimen.

The therapeutic benefits included not only achieving pCR and safe surgical margins, but also preserving swallowing and vocal functions. In this cohort, quality of life, swallowing function, and voice function improved after neoadjuvant therapy, transiently worsened during the radiotherapy phase, and gradually recovered from 6 months to 1 year after radiotherapy. These patterns suggest that the neoadjuvant-plus-surgery pathway did not cause persistent functional impairment, whereas radiotherapy remained the main period of functional deterioration (21).

Pathologic response and margin safety may also inform postoperative treatment intensity. In the present cohort, 4 patients underwent active surveillance, 2 received maintenance immunotherapy, 11 received radiotherapy for residual disease, and 3 received radiotherapy plus immunotherapy for high-risk pathological factors. ECOG-3311 showed that postoperative pathologic risk stratification could guide adjuvant treatment deintensification in HPV-related OPSCC (22). Our data suggested that responses after neoadjuvant therapy may provide a reference for individualized postoperative treatment in TSCC. This interpretation remains hypothesis-generating, because most patients in our analysis were p16-positive, and pCR or MPR does not necessarily translate into long-term survival benefit (36). Accordingly, the present findings predominantly reflect p16-positive disease and should be extrapolated cautiously to p16-negative TSCC. Notably, the only p16-negative patient achieved an overall ypT0N0 pCR after four cycles of neoadjuvant treatment and subsequently underwent R0 minimally invasive surgery, suggesting that this regimen may also have therapeutic potential in p16-negative TSCC. Standardized deintensification therefore requires longer follow-up and prospective validation.

Several limitations of the present study should be acknowledged. First, this was a single-center, single-arm exploratory study with a small sample size; therefore, the findings are intended for signal detection and effect estimation rather than superiority comparisons. Second, follow-up remains limited: at the data cutoff, follow-up ranged from 6 to 18 months, with no definite recurrence or death observed. These findings should be interpreted as preliminary early outcome signals rather than evidence of long-term survival benefit. Third, although this study focused on a single disease entity to reduce heterogeneity of primary sites, sufficient stratification according to p16/HPV status, PD-L1 expression, smoking and alcohol exposure, and differences in neoadjuvant cycles has not yet been performed; therefore, the identification of which patients are most likely to benefit from this regimen is still challenging.

Overall, the results of the present study suggested that triplet neoadjuvant therapy could improve short-term tumor shrinkage and the pathological response while allowing surgery to be performed smoothly with reliable margin safety. This strategy may help preserve normal tissue and related function to the greatest extent possible in patients and may also provide a basis for individualized adjustment of postoperative adjuvant treatment intensity. Given the generally favorable prognosis of patients with HPV-positive oropharyngeal cancer, mature overall survival data often require prolonged follow-up, and early efficacy signals may therefore be informative for the preliminary evaluation of treatment activity (37). However, whether these short-term advantages in efficacy can be translated into a clear long-term survival benefit still requires longer follow-up and higher-quality studies.

## Conclusions

In TSCC patients, the triplet neoadjuvant regimen comprising nimotuzumab, toripalimab, and chemotherapy resulted in a high radiological response rate, favorable pathological response, reliable margin safety, and good overall perioperative tolerability. In this cohort, individualized surgical planning based on both preneoadjuvant and postneoadjuvant assessments, together with intraoperative pathologic verification, allowed negative margins to be achieved in all patients. These findings suggest that postneoadjuvant tumor extent may inform the safe reduction in the extent of tumor resection without compromising R0 resection. Nevertheless, resection of the tonsillar primary lesion and cervical lymph node dissection remain necessary. These findings also provide preliminary evidence for the individualized adjustment of postoperative adjuvant treatment. Whether more patients can experience a survival benefit with this strategy requires further validation in large-scale, multicenter randomized controlled trials.

## Data Availability

The raw data supporting the conclusions of this article will be made available by the authors, without undue reservation.
